# Piezoelectric Nanofibrous Scaffolds for Promoting the Growth of Spiral Ganglion Neurons via Acoustic Stimulation

**DOI:** 10.1002/smmd.70055

**Published:** 2026-09-09

**Authors:** Dongyu Xu, Cuntu Cheng, Shan Xu, Chuan Bu, Shuangba He, Sizhe Song, Yu Wang, Busheng Tong, Yangnan Hu, Huan Wang, Renjie Chai

**Affiliations:** ^1^ Department of Otolaryngology Head and Neck Surgery Zhongda Hospital State Key Laboratory of Digital Medical Engineering Jiangsu Provincial Key Laboratory of Critical Care Medicine School of Life Sciences and Technology School of Medicine Advanced Institute for Life and Health Southeast University Nanjing China; ^2^ Department of Otolaryngology The First Hospital of China Medical University Shenyang China; ^3^ The Affiliated Lianyungang Hospital of Xuzhou Medical University The First People's Hospital of Lianyungang Lianyungang China; ^4^ The First Affiliated Hospital of Kangda College of Nanjing Medical University The First People's Hospital of Lianyungang Lianyungang China; ^5^ Department of Otorhinolaryngology Head and Neck Surgery School of Medicine Nanjing Tongren Hospital Southeast University Nanjing China; ^6^ Wenzhou Institute University of Chinese Academy of Sciences Wenzhou Zhejiang China; ^7^ Department of Otolaryngology, Head and Neck Surgery The First Affiliated Hospital of Anhui Medical University Hefei Anhui China; ^8^ School of Medical Engineering Affiliated Zhuhai People's Hospital Beijing Institute of Technology Zhuhai China; ^9^ The Eighth Affiliated Hospital Sun Yat‐sen University Shenzhen China; ^10^ Co‐Innovation Center of Neuroregeneration Nantong University Nantong China; ^11^ Department of Neurology Aerospace Center Hospital School of Life Science Beijing Institute of Technology Beijing China; ^12^ Department of Otolaryngology Head and Neck Surgery Sichuan Provincial People's Hospital School of Medicine University of Electronic Science and Technology of China Chengdu China; ^13^ Southeast University Shenzhen Research Institute Shenzhen China

**Keywords:** electrospinning, piezoelectric materials, spiral ganglion neurons, tissue‐engineering

## Abstract

The loss of spiral ganglion neurons (SGNs) and hair cells often leads to sensorineural hearing loss (SNHL), whose therapeutic effect depends on the quality and functional integrity of the residual SGNs. Compared with traditional pharmacological therapies, tissue engineering methods have been developed to provide a biomimetic environment for SGN regeneration, but static strategies and non‐responsive scaffolds still limit the therapy efficiency. Here, we present a composite piezoelectric nanofibrous scaffold responsive to acoustic stimulation to promote the growth of SGNs. The oriented nanofibrous scaffold comprising polyaniline (PANI), poly (L‐lactide) (PLLA) and gelatin was fabricated using electrospinning techniques. It was cytocompatible and provided a biomimetic culture environment with controllable electric signals for SGN regeneration. It has been confirmed that the piezoelectric nanofibrous scaffold could not only enhance cell adhesion and directional growth of SGNs, but also generate the electrical signals in response to acoustic stimulation, thereby accelerating the axon growth and functional development of SGNs. Therefore, the piezoelectric nanofibrous scaffolds could provide a new approach for SGN regeneration and a potential therapeutic strategy for SNHL.

## Highlights

- The incorporation of PANI into the piezoelectric nanofibrous scaffold significantly improves its piezoelectricity.
- The scaffold provides biomimetic components, oriented structures and electrical stimulation via acoustic excitation.
- Upon acoustic stimulation, the scaffold functions as an electroactive platform for promoting SGN growth and functional maturation.

## Graphical abstract

The piezoelectric nanofibrous scaffolds containing PANI, PLLA and Gelatin exhibited biomimetic structure, components, and piezoelectricity. Benefiting from the oriented nanofibers and electrical stimulation induced by wireless acoustic excitation, the SGNs cultured on the scaffolds showed enhanced neurite alignment, extension, and growth cone development, indicating that the electrical stimulation induced by acoustic excitation significantly promoted SGN development.

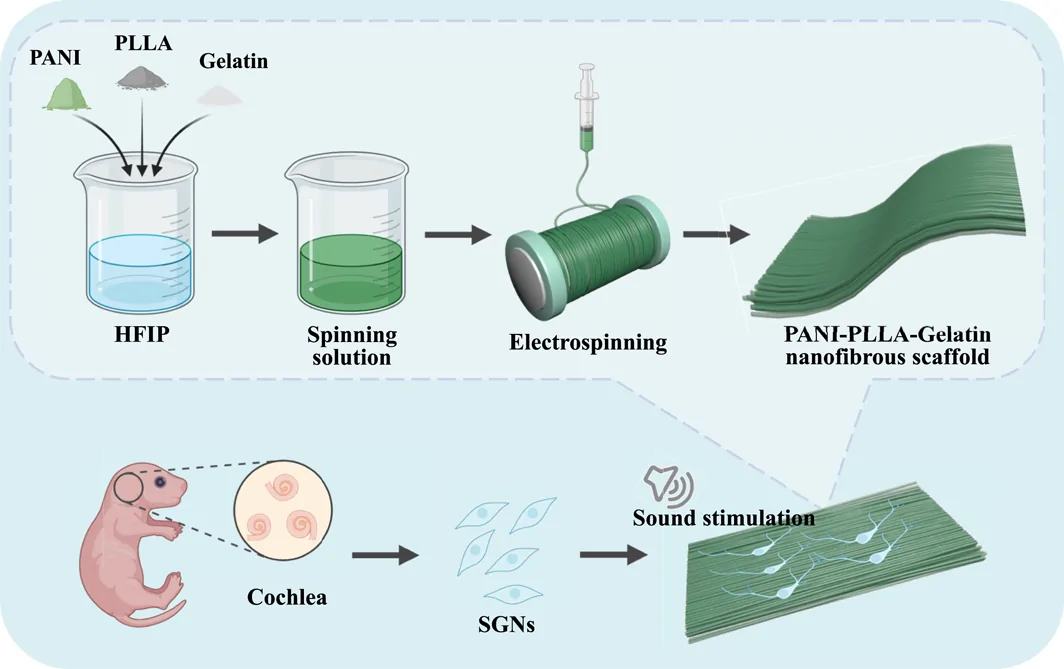

## 1 Introduction

Sensorineural hearing loss (SNHL) is a prevalent global health concern, primarily caused by the degeneration or loss of spiral ganglion neurons (SGNs) and hair cells [1, 2, 3, 4, 5, 6, 7]. As the SGNs play a key role in perceiving the electrical signals from inner ear hair cells and transmitting them into the brain to produce hearing, the regeneration and functional repair of SGNs are significant in SNHL treatment, which remains challenging for the independent drug treatment [8, 9]. Recently, diverse approaches have been developed to promote the SGN regeneration, involving the conductive substrates with pattern surfaces, polymer nanofibers, gene therapy, and stem cell transplantation [10, 11, 12, 13, 14, 15, 16, 17]. Among them, the electrospun nanofibers possess an oriented structure that mimics the natural extracellular matrix, promoting cell adhesion, proliferation and directed growth [11, 18, 19, 20]. Although much progress has been achieved, the developed static strategies and unresponsive scaffolds can not provide stimulation to SGNs, which limit the regeneration effect of SGNs. Therefore, a novel, responsive bio‐scaffold capable of dynamically stimulating SGNs to achieve enhanced regeneration and protection is still anticipated.

Here, we present a piezoelectric nanofibrous scaffold responsive to acoustic stimulation for promoting the growth and development of SGNs. Piezoelectric materials exhibit electromechanical properties by transforming mechanical force into electric polarization without an external power supply, in addition to good biocompatibility, flexibility and mechanical robustness, and these inherent properties contribute to their potential for eliciting specific cellular responses [21, 22, 23, 24, 25, 26, 27]. Meanwhile, local surface charges generated by bioelectric fields induce nerve cells to secrete various growth factors and promote the growth of nerve fibers, with depolarizing electrical stimulation producing a similar effect [28, 29, 30]. Consequently, the composite piezoelectric nanofibers have been confirmed to be capable of promoting rapid nerve regeneration and increasing expression of nerve growth factor due to the local piezoelectric effect [29, 31]. Moreover, the piezoelectric properties can be significantly enhanced through processing techniques such as electrospinning, which simultaneously aligns the inherent piezoelectric dipoles and controls the fiber morphology [32, 33, 34]. Therefore, the integration of these controllable piezoelectric properties into electrospun nanofibers is anticipated to provide dynamic electrical stimulation, offering a promising solution to the current challenges in SGN regeneration.

**FIGURE 1:**
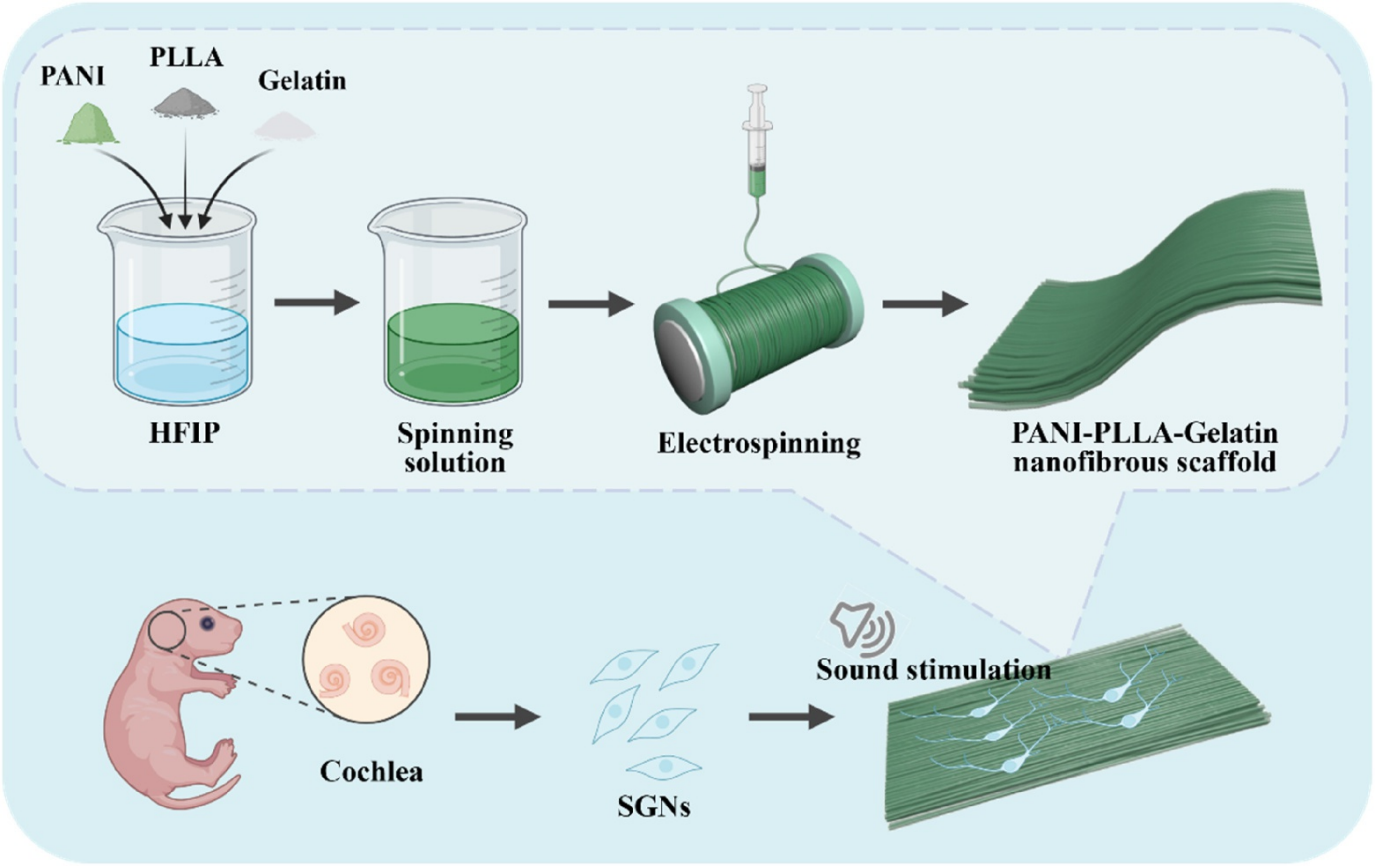
Schematic illustration of the fabrication of piezoelectric electrospun nanofibrous scaffolds and their interaction with SGNs via sound stimulation.

In this paper, we manufactured the desired piezoelectric electrospun nanofibrous scaffold composed of polyaniline (PANI), poly (L‐lactide) (PLLA) and gelatin for promoting the growth of SGNs, as illustrated in Figure 1. Benefiting from the piezoelectricity of PLLA and locally enhanced electrical field induced by conductive PANI, the PANI‐PLLA‐Gelatin electrospun nanofibrous scaffold exhibited good piezoelectric performance and generated corresponding electric potential changes via acoustic stimulation. In addition, gelatin was incorporated to mimic type IV collagen, a key component of the cochlear basement membrane, thereby endowing the scaffold with excellent cytocompatibility. Therefore, this piezoelectric electrospun nanofibrous scaffold provided a bionic culture environment with safe and controllable electric signals for SGN regeneration. The SGNs cultured on the scaffolds displayed good adhesive and oriented growth. More importantly, the electrical signals of the scaffolds under acoustic stimulation could promote the growth and functional maturation of SGNs, causing longer axons and stronger growth cone development. Compared with static culture, the acoustic mechanical cues and piezoelectric cues provide dynamic physical cues that help maintain SGN growth and functional activity. Therefore, the PANI‐PLLA‐Gelatin piezoelectric electrospun nanofibrous scaffold shows potential in promoting the growth and regeneration of SGNs, with potential application in SNHL treatment.

## 2 Results and Discussion

**FIGURE 2:**
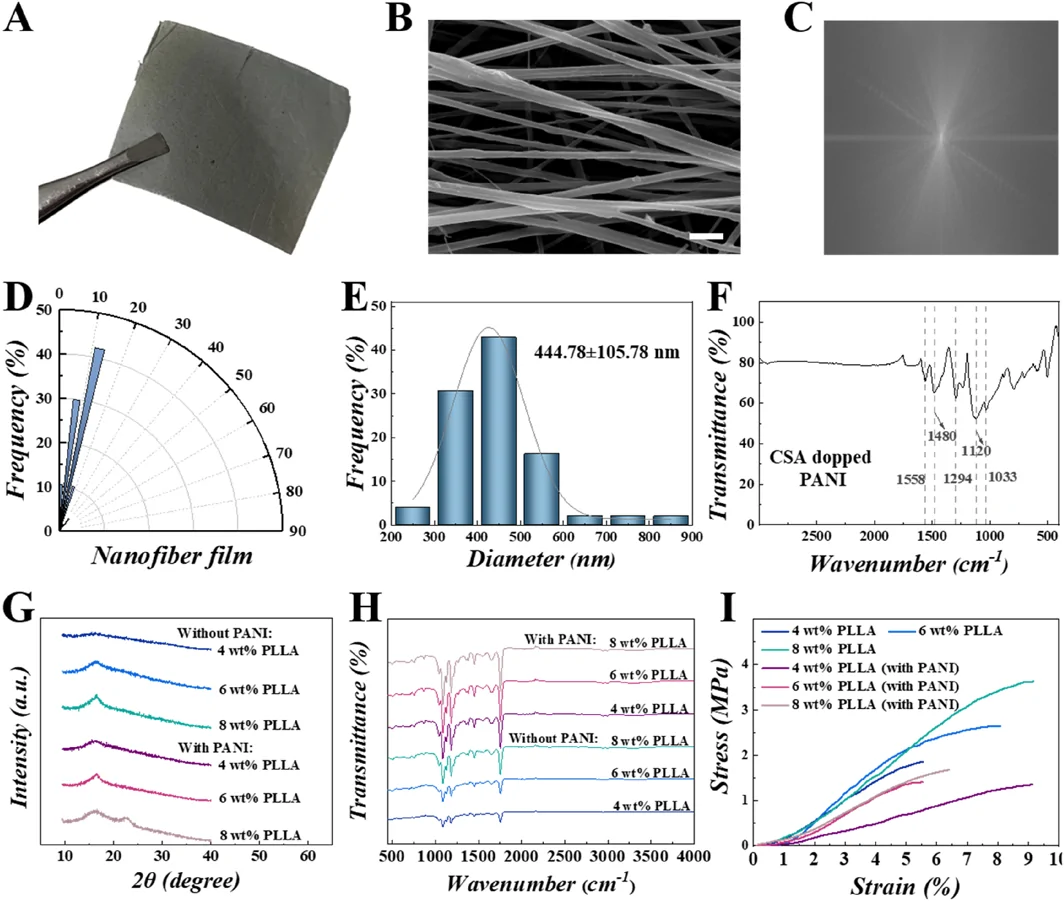
Morphology and composition of the piezoelectric electrospun nanofibrous scaffold. (A) Photo of the PANI‐PLLA‐Gelatin piezoelectric electrospun nanofibrous scaffold. (B) SEM image of the PANI‐PLLA‐Gelatin piezoelectric electrospun nanofibrous scaffold. (C) FFT result of the SEM image. (D) Polar histograms of nanofiber orientation. (E) Statistical results of nanofiber diameter distribution. (F) FTIR spectrum of PANI‐CSA powder. (G–I) XRD results (G), FTIR spectra (H) and tensile stress‐strain curves (I) of the piezoelectric electrospun nanofibrous scaffolds with different compositions. Scale bar is 3 μm.

In a typical experiment, the PANI‐PLLA‐Gelatin nanofibrous scaffolds were prepared by roller‐assisted electrospinning (Figure 2A). The polymer solution could form liquid jets under a high‐voltage electric field to prepare nanoscale continuous fibers, while the mechanical stretching provided by a roller can impart alignment to the resulting nanofibers. Further investigation into the effect of roller speed on nanofiber morphology revealed that higher speeds enhanced nanofiber alignment (Supporting Information S1: Figure S1). As shown in Figure 2B,E, the PANI‐PLLA‐Gelatin nanofibrous scaffold exhibited relatively fine fiber diameters under the polarized stretching process, with an average diameter of approximately 445 nm. The Fast Fourier transform (FFT) result in Figure 2C and orientation frequency in Figure 2D showed the consistent alignment in the nanofibrous scaffold, which is the physical guidance cue within a biomimetic physiological environment. In contrast, the FFT result of PANI‐PLLA‐Gelatin nanofibers collected at a low rotating drum speed showed their random distribution (Supporting Information S1: Figure S2). PANI was modified by camphorsulfonic acid (CSA) doping, thus the intramolecular and intermolecular conformation of PANI was more conducive to the delocalization of charge on the molecular chain [35]. Fourier Transform Infrared spectroscopy (FTIR) results showed the C‐C bending vibration (1120 cm^−1^) in the benzene ring, N=Q=N (*Q* referred to the quinone ring) characteristic absorption peak (1294 cm^−1^), C‐N stretching vibration (1480 cm^−1^), and skeleton vibration stretching vibration (1558 cm^−1^) of quinone and benzene structure on the polyaniline chain, indicating the successful doping of PANI and CSA (Figure 2F). Besides, the modified PANI changed from white to dark green after electroactivation, demonstrating the addition of PANI within the scaffold (Figure 2A). As shown in Supporting Information S1: Figure S3, the elemental mapping of sulfur (S) obtained by energy‐dispersive X‐ray spectroscopy (EDS) revealed the distribution of CSA‐doped PANI within the nanofibers, while the transmission electron microscopy (TEM) image further demonstrated the incorporation of PANI inside the nanofiber.

To further explore the impacts of PLLA and PANI on the component and responsiveness of nanofibrous scaffold, the piezoelectric electrospun nanofibrous scaffolds obtained from 4%, 6% and 8% PLLA solution with or without PANI were prepared. The X‐ray diffraction (XRD) spectra shown in Figure 2G demonstrated that the PANI‐doped PLLA nanofibrous scaffold showed typical 2θ peak at 16.4°, similar to the PLLA nanofibers, indicating the crystalline structure of PLLA in the PANI‐PLLA‐Gelatin nanofibrous scaffolds. The FTIR spectra in Figure 2H showed the characteristic peaks of PLLA at 1755, 1182 and 1087 cm^−1^, which were responsible for the piezoelectricity in the PLLA nanofibers. These results, with the XRD results, suggested changes in the crystalline structure of PLLA. Besides, the mechanical properties of the nanofibrous scaffolds are indispensable for supporting the adhesion and growth of cells. The effect of PANI and concentration of PLLA on the tensile properties of nanofibrous scaffolds was explored by tensile testing (Figure 2I). The increase of concentration of PLLA resulted in the substantial increase in tensile strength of the nanofibrous scaffolds, while the incorporation of PANI reduces the tensile strength of the scaffolds. This may be attributable to the heterogeneity of PANI doping, which compromised the mechanical strength. After balancing the processability, mechanical properties, and electrical characteristics, we selected the composition of 2% PANI—6% PLLA—2% Gelatin for the subsequent fabrication of nanofibrous scaffolds.

**FIGURE 3:**
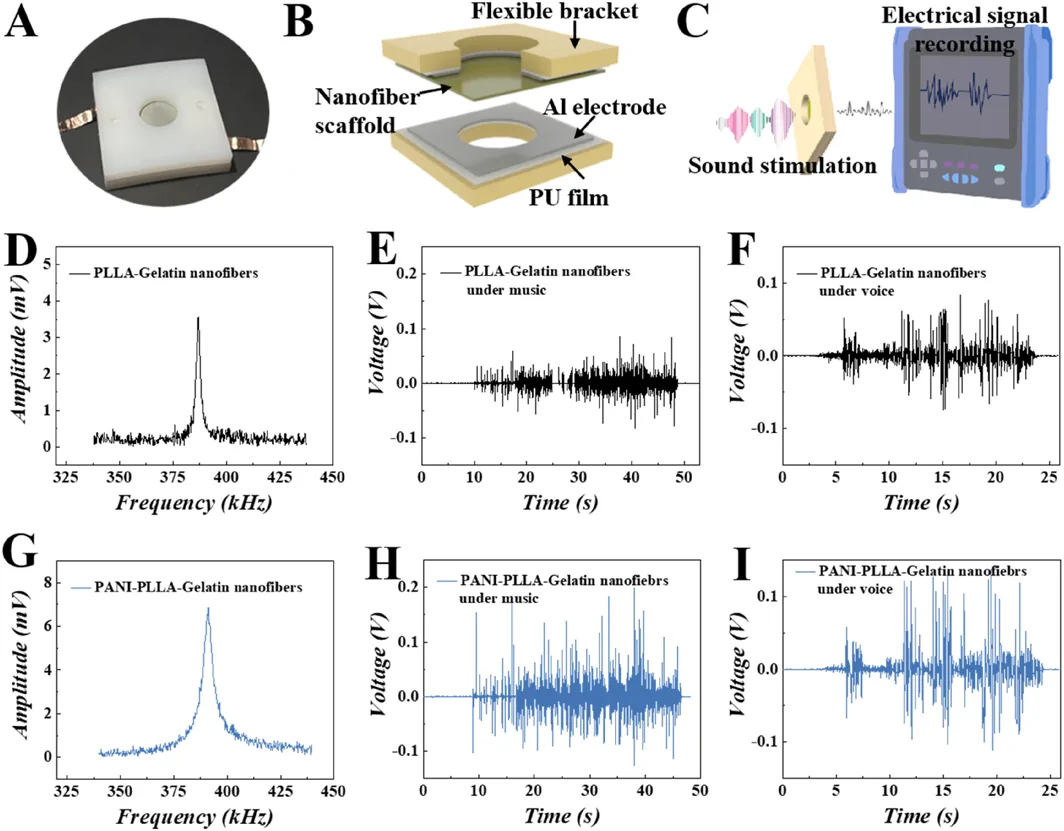
Piezoelectric characterization of the piezoelectric nanofibrous scaffold. (A) Photo of the experimental setup for piezoelectric characterization. (B, C) Schematic illustration of the experimental setup (B) and the piezoelectric performance test system (C). (D, G) Piezoresponse of the PLLA‐Gelatin nanofibers (D) and the PANI‐PLLA‐Gelatin nanofibers (G) versus excitation frequency (Vac: 1 V). (E, H) Open‐circuit voltage output generated by the PLLA‐Gelatin nanofibers (E) and the PANI‐PLLA‐Gelatin nanofibers (H) under music. (F, I) Open‐circuit voltage output generated by the PLLA‐Gelatin nanofibers (F) and the PANI‐PLLA‐Gelatin nanofibers (I) under human voice recording.

Subsequently, we investigated the piezoelectric properties of the nanofibrous scaffold. As illustrated in Figure 3A,B, the piezoelectric nanofiber scaffold was sandwiched between two aluminum foil electrodes and PU flexible films, and further was fixed within a flexible bracket featuring a central aperture. The piezoelectric signal output of the nanofibrous scaffold under acoustic waves was recorded by an electrical signal recording system (Figure 3C). Before that, the piezoelectric response of nanofibers with and without PANI doping was first evaluated by piezo‐response force microscopy (PFM). As shown in the frequency‐dependent piezoresponse analysis, PANI‐PLLA‐Gelatin nanofibers showed higher piezoelectric activity than PLLA‐Gelatin nanofibers with the increased peak amplitude in Figure 3D,G. This result revealed that the conductive elements in the polymer chains may enhance the local electric field during the electrospinning process, thereby improving the PLLA chain polarization and piezoelectricity [36]. More importantly, the piezoelectric nanofibrous scaffolds demonstrated acoustic‐driven piezoelectric output under both music (Figure 3E,H) and voice stimulation (Figure 3F,I). The results showed that the piezoelectric nanofibrous scaffolds generated open‐circuit voltage output in response to acoustic stimulation, and the piezoelectric output was significantly enhanced by PANI doping. The piezoelectric output generated from the piezoelectric nanofibrous scaffolds restored the characteristics of the sound waveforms (Supporting Information S1: Figure S4).

To improve the biocompatibility of the nanofibrous scaffolds for cell culture, the scaffolds were treated by plasma (Supporting Information S1: Figure S5). NIH 3T3 cells were cultured on the piezoelectric nanofibrous scaffolds and plates (Control group), respectively, and then the live cells were labeled with calcein‐AM from day 1 to day 5. The results (Supporting Information S1: Figure S6) showed that the NIH 3T3 cells were well attached on the scaffolds, and exhibited better oriented growth compared to the Control group, indicating that the components of the scaffold not only have no toxicity to NIH 3T3 cell's growth and proliferation but also guide the orientation. Furthermore, the SGNs were cultured on coverslips (Control group) and on PANI‐PLLA‐Gelatin nanofibrous scaffolds with acoustic stimulation (Scaffold + Sound group). The live/dead cell staining images indicated that the SGNs exhibited good adhesion and axonal extension in the Scaffold + Sound group, suggesting that the applied mechanoelectrical stimulation is biocompatible (Supporting Information S1: Figure S7).

**FIGURE 4:**
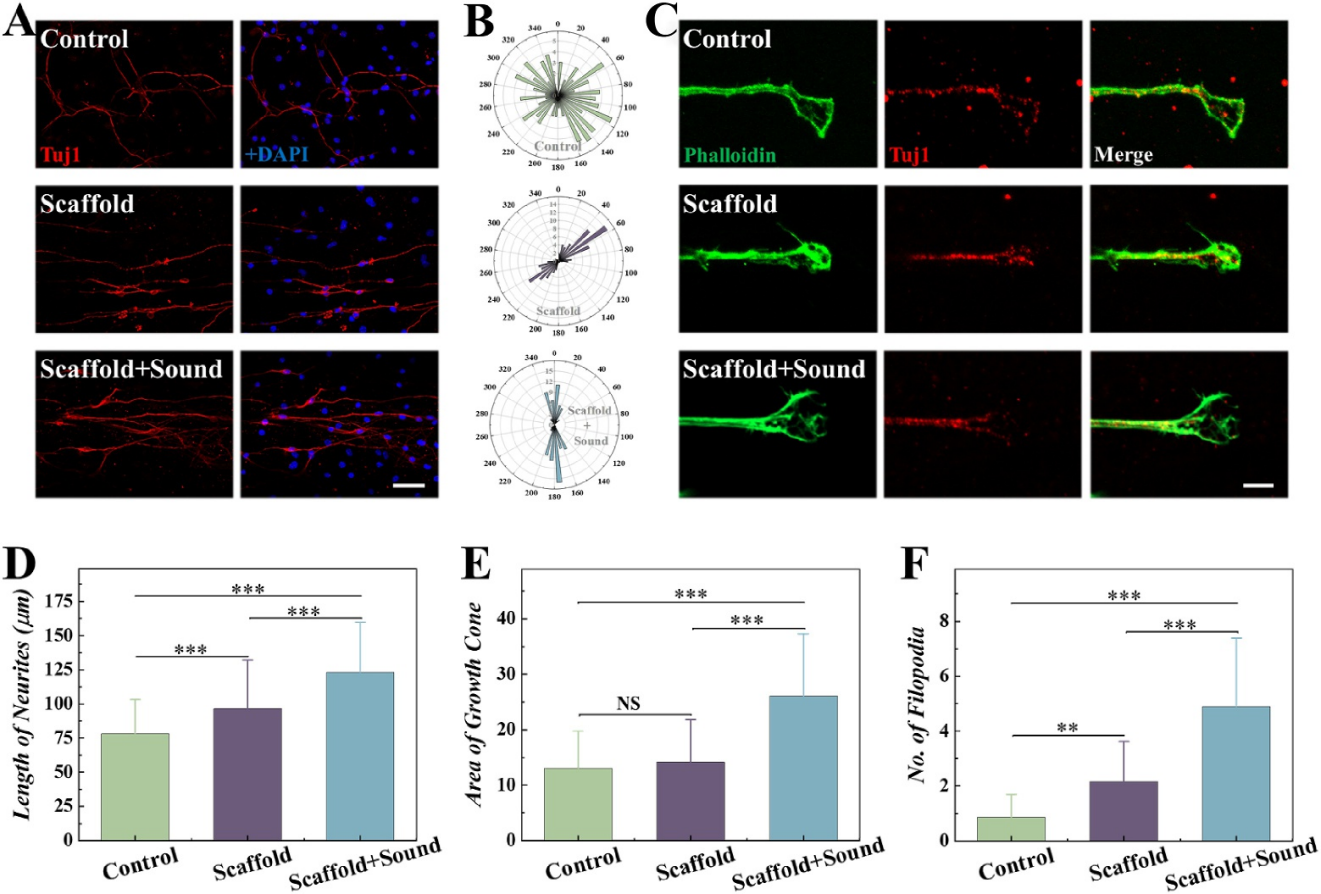
Effect of the scaffold under acoustic stimulation on the growth of SGNs. (A) Representative immunofluorescence images of SGNs cultured in the control group (top), scaffold (middle), scaffold with sound stimulation (bottom). SGNs were stained by Tuj1 (red), and nuclei were stained by DAPI (blue). (B) Orientation distribution of SGN axons. (C) Immunofluorescence images of SGN's growth cones (phalloidin: green; Tuj1: red). (D–F) Statistical results of SGN neurite length (D), growth cone area (μm^2^) (E) and number of filopodia (F). Scale bars are 50 μm in (A) and 5 μm in (C).

Based on such nanofibrous scaffolds, we further investigated the effect of the mechanoelectrical stimulation on the growth and development of SGNs. The SGNs were cultured on different substrates with different treatment: glass coverslips (Control group), PANI‐PLLA‐Gelatin nanofibrous scaffolds (Scaffold group) and PANI‐PLLA‐Gelatin nanofibrous scaffolds with daily sound stimulation (Scaffold + Sound group). Following the 7‐day treatment, the SGN morphology, neurite extension, and growth cones were evaluated by immunofluorescence staining for the neuronal marker *β*‐III tubulin (Tuj1) and F‐actin. The confocal fluorescence images and the statistical results of the neurite length of the SGNs indicated that the mechanoelectrical effect of the piezoelectric nanofibrous scaffold significantly promoted the outgrowth of neurites (Figure 4A,D). Besides, in contrast to the randomly oriented neurites in the control group, the SGNs on the nanofibrous scaffolds exhibited neurite extension aligned with the nanofiber orientation, which was attributed to the aligned topographical cues (Figure 4B). Growth cones serve as the primary interface for neurons to sense and respond to topographical, chemical, and biophysical signals, thereby directing functional connectivity. As shown in Figure 4C, growth cones in the Scaffold + Sound group exhibited a more advanced developmental state compared to the Control group. Quantitative analysis of growth cone area and filopodia number further confirmed that the mechanoelectrical stimulation generated by the PANI‐PLLA‐Gelatin nanofibrous scaffold under acoustic stimulation promoted growth cone development and the functional maturation of SGNs (Figure 4E,F).

**FIGURE 5:**
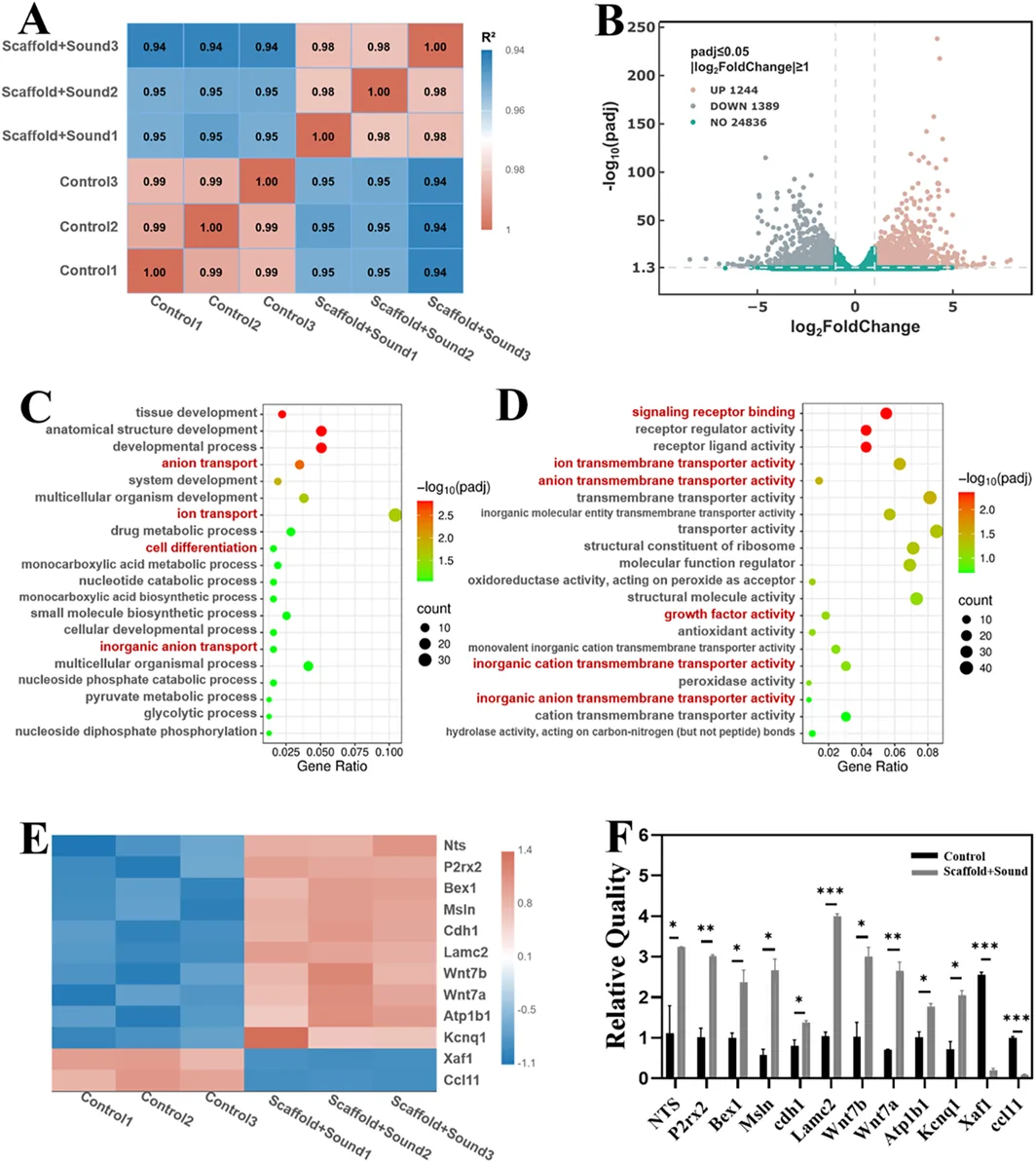
Differential gene analysis of SGNs in the PANI‐PLLA‐gelatin nanofibrous scaffold with sound stimulation (scaffold + sound) and Control groups. (A) Heat map showing the gene expression level correlation between samples. (B) Volcano plot displaying differentially expressed genes between the scaffold + sound and Control groups. The horizontal axis represents the change of gene expression multiple between the two groups (log_2_FoldChange), and the vertical axis represents the significance level of the expression difference between the two groups (−log_10_padj). |log_2_FoldChange| ≥ 1 and padj ≤ 0.05 were the screening standards for differential genes. Up‐regulated genes are shown as pink dots (right), down‐regulated genes are shown as gray dots (left), and genes with no difference are shown as green dots. (C, D) GO enrichment analysis results for BP (C) and MF (D) categories. (E) Cluster heat maps of differentially expressed genes in the Scaffold + Sound and Control groups. (F) mRNA expression levels of genes in the cluster heat map.

We extracted mRNA from SGNs in the control group and scaffold + Sound group, and performed RNA sequencing (RNA‐seq). Firstly, the squared Pearson correlation coefficient (*R* ^2^) of samples through FPKM values of all genes in each sample is shown in the heat map (Figure 5A). The results suggested that FPKM values of all annotated genes revealed high reproducibility across biological replicates with all pairwise *R* ^2^ ≥ 0.94. *R* ^2^ of samples within groups was greater than that between them, demonstrating high similarity of expression patterns among replicates, robust experimental consistency, and reliability of biological repetition. As shown in Supporting Information S1: Figure S8 and Figure 5B, the mechanoelectrical stimulation generated by the acoustic‐driven piezoelectric nanofibrous scaffold could regulate the endogenous gene expression in SGNs. Differential gene expression analysis in the volcano plot identified 1244 up‐regulated and 1389 down‐regulated genes in the Scaffold + Sound group compared with the Control group. Gene ontology (GO) enrichment analysis revealed that the significantly enriched biological processes (BPs) were primarily associated with developmental processes and ion transport (Figure 5C). Meanwhile, the enriched molecular functions (MFs) were mainly related to the regulation of the growth factor activity and the ion channel activity (Figure 5D).

Hierarchical clustering of 12 significantly differentially expressed genes demonstrated distinct expression patterns: *Nts*, *P2rx2*, *Bex1*, *Msln*, *Cdh1*, *Lamc2*, *Wnt7b*, *Wnt7a*, *Atp1b1*, and *Kcnq1* were up‐regulated in the Scaffold + Sound group, whereas *Xaf1* and *Ccl11* showed down‐regulation (Figure 5E). We further performed qPCR experiments to validate the mRNA expression levels of the 12 selected differentially expressed genes, and the results were consistent with the clustering results (Figure 5F). The differentially expressed genes were significantly enriched in neuronal electrical activity, axon guidance, and neurotrophic support. In addition, the calcium oscillation tracer waveform of SGNs in the Scaffold + Sound group showed more frequent calcium oscillations than that in the Control group, indicating the increased activity of calcium channel proteins (Supporting Information S1: Figure S9). The KEGG pathway enrichment results also confirmed the activation of key pathways in SGNs, including extracellular matrix remodeling, cell adhesion, cytokine‐cytokine receptor interaction, and calcium signaling (Supporting Information S1: Figure S10). Therefore, the piezoelectric nanofibrous scaffold with acoustic stimulation system potentially modulated SGNs through multiple synergistic mechanisms. Specifically, the mechanoelectrical stimulation may promote neuritogenesis and SGN maturation. Furthermore, the coordinated expression changes in ion transport and calcium signaling pathway imply improved cell signal communication caused by the piezoelectric scaffold.

## 3 Conclusion

In summary, we have successfully prepared a composite piezoelectric nanofibrous scaffold that responded to acoustic stimulation. The oriented nanofibers of the scaffold provide a bionic microenvironment for the cells, while the acoustic‐driven mechanoelectrical stimulation modulates the behavior of SGNs. In vitro evaluation experiment validated the cytocompatibility of composite piezoelectric nanofibrous scaffold, and demonstrated that the scaffold could guide directional growth of SGNs. Furthermore, the introduction of the microstructure and mechanoelectrical stimulation generated by the scaffold with acoustic stimulation could promote the development of axon and growth cone. These effects were associated with increased expression of genes related to voltage‐gated channels, ion transport, and axon growth. Thus, the piezoelectric nanofibrous scaffold with topographical and electroactive cues promotes SGN growth and maturation, potentially inhibiting SGN degeneration and offering a promising new therapeutic intervention for SNHL.

## 4 Methods

### 4.1 Materials

Polyaniline (PANI) and poly (L‐lactide) (PLLA) were purchased from Merck. Gelatin was from Sigma. Camphorsulfonic acid (CSA), chloroform, and hexafluoroisopropanol (HFIP) were bought from Shanghai Yeasen Biotechnology Co. Ltd. Anti‐βIII‐tubulin antibody (Tuj1) and phalloidin‐iFluor 488 reagent were bought from Abcam Co. Ltd. 4% paraformaldehyde (PFA) fix solution, 5% BSA blocking buffer, DAPI, Triton X‐100, calcein‐AM, and propidium iodide (PI) were obtained from Beyotime Co. Ltd.

### 4.2 Preparation for Electrospinning Solution

Firstly, PANI and CSA were mixed at a 1:1 mass ratio and dissolved in chloroform. The solution was stirred at 4°C for 24 h to obtain a CSA‐PANI solution. Subsequently, the resulting mixture was thoroughly washed with anhydrous ethanol, collected via suction filtration, and dried to obtain CSA‐PANI powder for future use. Electrospinning solutions were prepared by dissolving CSA‐PANI, PLLA, and gelatin in HFIP under continuous stirring for 24 h. The concentration of CSA‐PANI was set at 0 or 2 wt%, and that of gelatin was fixed at 2 wt%. The PLLA concentration was varied among 4, 6, and 8 wt% to formulate different compositions. The obtained nanofibers were named as PLLA‐Gelatin nanofibers (without CSA‐PANI) and PANI‐PLLA‐Gelatin nanofibers (with CSA‐PANI).

### 4.3 Preparation of Piezoelectric Nanofibrous Scaffolds

In this study, an electrospinning setup with a rotating drum was used. The electrospinning solution was fed at a rate of 0.8 mL/h through a 20 G stainless steel needle under an applied voltage of 14 kV. The resulting polymer jet was collected by a rotating drum collector. Following electrospinning, the obtained nanofibrous scaffolds were dried for 24 hours to allow for complete solvent evaporation.

### 4.4 Experimental Setup for Piezoelectric Characterization

The PANI‐PLLA‐Gelatin nanofibrous scaffold was sequentially assembled into a layered structure with the scaffold as the core, sandwiched between two aluminum foil electrodes, which were in turn enclosed by two PU flexible films, and two flexible brackets. This assembly featured a central aperture (2 cm in diameter) for acoustic wave exposure. The generated open‐circuit voltage of the PANI‐PLLA‐Gelatin nanofibrous scaffold was recorded by a memory hicorder (HIOKI, MR8875‐30). Besides, the piezoresponse of the nanofibrous scaffold was tested by PFM (MFP‐3D‐SA).

### 4.5 Cytocompatibility Test

Piezoelectric nanofibrous scaffolds were sterilized by UV light and then stored for future use. NIH 3T3 cells were cultured on plate and piezoelectric scaffolds for 5 days. On days 1, 3, and 5, the NIH 3T3 cells were stained by 0.1 vol% calcein‐AM and PI to indicate live and dead cells, respectively. The stained cells were then observed and imaged using a fluorescence microscope.

### 4.6 SGN Culture and Characterization

Primary SGNs were cultured under three conditions: round coverslips (Control), the piezoelectric nanofibrous scaffolds (Scaffold), and the piezoelectric nanofibrous scaffolds with acoustic stimulation (Scaffold + Sound). For the Scaffold + Sound group, the scaffolds with SGNs were moved to a flexible‐bottom plate 24 h post‐seeding and exposed to sound stimulation for 30 min each day (100 dB SPL, 2 cm between the plate and the sound source). On day 7, all samples were harvested and fixed in 4% PFA for 50 min. The samples were immunostained for the neuronal marker Tuj1, followed by counterstaining with phalloidin and DAPI. Confocal fluorescence microscope was finally used to observe and record the cells. For RNA sequencing (RNA‐seq) analysis, total RNA was extracted using Trizol reagent on day 14 and submitted for sequencing at NovoMagic Co. Ltd (Shanghai NewCore Biotechnology Co. Ltd.).

### 4.7 Statistical Analysis

Fiji was used to analyze SEM images and fluorescence images. All experimental data were expressed as mean ± standard deviation (SD), with *n* ≥ 3. Significant difference was analyzed by Kruskal–Wallis test and was indicated as follows: *p ≤ 0.05, **p ≤ 0.01, ***p ≤ 0.001; ns, not significant.

## Author Contributions

D.X. and C.C. contributed equally to this work. R.C., Y.H., and H.W. conceived the idea. C.C. conducted experiments. C.C. and D.X. carried out data analysis and wrote the manuscript. Y.W., B.T., Y.H., and H.W. assisted with paper writing. S.X., C.B., S.H., and S.S. contributed to the scientific discussion of the work. S.S. assisted with the revision of the manuscript.

## Ethics Statement

All mice procedures were approved by the Animal Experimental Ethical Inspection Committee of Southeast University.

## Conflicts of Interest

Renjie Chai is an associate editor of *Smart Medicine* and was not involved in the editorial review or the decision to publish this article. All authors declare that there are no competing interests.

## Supporting information

Supporting Information S1

## Data Availability

The data that support the findings of this study are available in the supplementary material of this article.
