# Supplementary material for: Piezoelectric Nanofibrous Scaffolds for Promoting the Growth of Spiral Ganglion Neurons via Acoustic Stimulation
Source: Smart Med. 2026 Sep 9;5(5):e70055. doi: 10.1002/smmd.70055 (PMC13573972; doi:10.1002/smmd.70055)
Supplement: Supplementary file 1 [file SMMD-5-e70055-s001.docx]

Supporting Information

Piezoelectric nanofibrous scaffolds for promoting the growth of spiral ganglion neurons via acoustic stimulation

Dongyu Xu ^1#^, Cuntu Cheng ^1#^, Shan Xu^2^, Chuan Bu^3,4^, Shuangba He^5^, Sizhe Song^1^, Yu Wang ^6,*^, Busheng Tong^7,^*, Yangnan Hu^8,^*, Huan Wang ^9,*^, Renjie Chai ^1,10,11,12,13,*^

^1^ Department of Otolaryngology Head and Neck Surgery, Zhongda Hospital, State Key Laboratory of Digital Medical Engineering, Jiangsu Provincial Key Laboratory of Critical Care Medicine, School of Life Sciences and Technology, School of Medicine, Advanced Institute for Life and Health, Southeast University, Nanjing 210096, China

^2^ Department of Otolaryngology, The First Hospital of China Medical University, Shenyang 110001, China

^3^ The Affiliated Lianyungang Hospital of Xuzhou Medical University/ The First People's Hospital of Lianyungang, China

^4^ The First Affiliated Hospital of Kangda College of Nanjing Medical University/The First People's Hospital of Lianyungang, China

^5^ Department of Otorhinolaryngology Head and Neck Surgery, School of Medicine, Nanjing Tongren Hospital, Southeast University, Nanjing, China

^6^ Wenzhou Institute, University of Chinese Academy of Sciences, Wenzhou, Zhejiang 325001, China

^7^ Department of Otolaryngology, Head and Neck Surgery, The First Affiliated Hospital of Anhui Medical University, 218 Jixi Road, Hefei, Anhui,

230031, China

^8^ School of Medical Engineering, Affiliated Zhuhai People’s Hospital, Beijing Institute of Technology, Zhuhai, 519088, China

^9^ The Eighth Affiliated Hospital, Sun Yat-sen University, Shenzhen 518033, China.

^10^ Co-Innovation Center of Neuroregeneration, Nantong University, Nantong 226001, China

^11^ Department of Neurology, Aerospace Center Hospital, School of Life Science, Beijing Institute of Technology, Beijing 100081, China

^12^ Department of Otolaryngology Head and Neck Surgery, Sichuan Provincial People's Hospital, School of Medicine, University of Electronic Science and Technology of China, Chengdu 610072, China

^13^ Southeast University Shenzhen Research Institute, Shenzhen 518063, China

Email: [yuwang@wiucas.ac.cn](mailto:yuwang@wiucas.ac.cn) (Yu Wang); [tongbusheng@ahmu.edu.cn](mailto:tongbusheng@ahmu.edu.cn) (Busheng Tong); [yangnanhu@bit.edu.cn](mailto:yangnanhu@bit.edu.cn) (Yangnan Hu); [wangh679@mail.sysu.edu.cn](mailto:wangh679@mail.sysu.edu.cn) (Huan Wang); renjiec@seu.edu.cn (Renjie Chai)

**Figure S1.** SEM images of PANI-PLLA-Gelatin nanofibers collected at different rotating drum speeds. (a-f): speeds of 2700, 2800, 2900, 3000, 3100 and 3200 rpm. Scar bar is 2 μm.

**Figure S2.** FFT result of the SEM image of PANI-PLLA-Gelatin nanofibers collected at 2700 rpm.

**Figure S3.** EDS mapping images and TEM image of the PANI-PLLA-Gelatin nanofibers.

**Figure S4.** (a) Piezoelectric output of the PANI-PLLA-Gelatin nanofibers under music stimulation. (b) Time-domain waveform and (c) spectrogram of music.

**Figure S5.** Water contact angle images (a) and corresponding statistical analysis (b) of the nanofibrous scaffold before and after plasma hydrophilic treatment.

**Figure S6.** Representative fluorescence images of NIH 3T3 cells cultured on nanofibrous scaffold and plate (Control) labeled with Calcein-AM (green). Scale bar is 20 μm.

**Figure S7.** Live/dead staining images of SGNs cultured on coverslips (Control) (a) and the nanofibrous scaffold with sound stimulation (Scaffold+Sound) (b). Scale bar is 20 μm.

**Figure S8.** Venn diagram of co-expressed SGN genes between Control and Scaffold+Sound groups.

**Figure S9.** Calcium oscillation tracer waveforms of SGNs in (a) the Control group and (b) the Scaffold+Sound group.

**Figure S10.** KEGG pathway enrichment results.
